# Catalpol Attenuates Oxidative Stress and Inflammation via Mechanisms Involving Sirtuin-1 Activation and NF-κB Inhibition in Experimentally-Induced Chronic Kidney Disease

**DOI:** 10.3390/nu15010237

**Published:** 2023-01-03

**Authors:** Nur Elena Zaaba, Suhail Al-Salam, Sumaya Beegam, Ozaz Elzaki, Javed Yasin, Abderrahim Nemmar

**Affiliations:** 1 Department of Physiology, College of Medicine and Health Sciences, United Arab Emirates University, Al-Ain P.O. Box 15551, United Arab Emirates; 2 Department of Pathology, College of Medicine and Health Sciences, United Arab Emirates University, Al-Ain P.O. Box 15551, United Arab Emirates; 3 Department of Internal Medicine, College of Medicine and Health Sciences, United Arab Emirates University, Al-Ain P.O. Box 15551, United Arab Emirates

**Keywords:** catalpol, chronic kidney disease, adenine, inflammation, oxidative stress, apoptosis, DNA damage, sirtuin-1

## Abstract

Chronic kidney disease (CKD) is a stealthy disease, and its development is linked to mechanisms including inflammation and oxidative stress. Catalpol (CAT), an iridoid glucoside from the root of *Rehmannia glutinosa*, is reported to manifest anti-inflammatory, antioxidant, antiapoptotic and antifibrotic properties. Hence, we studied the possible nephroprotective effects of CAT and its mechanisms in an adenine-induced (0.2% *w*/*w* in feed for 4 weeks) murine model of CKD by administering 5 mg/kg CAT to BALB/c mice for the duration of 4 weeks except during weekends. Upon sacrifice, the kidney, plasma and urine were collected and various physiological, biochemical and histological endpoints were assessed. CAT significantly ameliorated the adenine-induced altered body and kidney weight, water intake, urine volume, and concentrations of urea and creatinine in plasma, as well as the creatinine clearance and the albumin and creatinine ratio. Moreover, CAT significantly ameliorated the effect of adenine-induced kidney injury by reducing the kidney injury molecule-1, neutrophil gelatinase-associated lipocalin, cystatin C and adiponectin. Similarly, the augmented concentrations of markers of inflammation and oxidative stress in the adenine-treated group were markedly reduced with CAT pretreatment. Furthermore, CAT prevented adenine-induced deoxyribonucleic acid damage and apoptotic activity in the kidneys. Histologically, CAT significantly reduced the formation of tubular necrosis and dilation, as well as interstitial fibrosis in the kidney. In addition to that, CAT significantly decreased the adenine-induced increase in the phosphorylated NF-κB and reversed the reduced expression of sirtuin-1 in the kidney. In conclusion, CAT exhibits salutary effects against adenine-induced CKD in mice by mitigating inflammation, oxidative stress and fibrosis via mechanisms involving sirtuin-1 activation and NF-κB inhibition. Confirmatory studies are warranted in order to consider CAT as a potent nephroprotective agent against CKD.

## 1. Introduction

Cases of chronic kidney disease (CKD) have reached an epidemic level, affecting one in every ten people globally. Without effective interventions, CKD can advance to end-stage renal failure (ESRF), which will significantly increase the risk of premature death unless kidney replacement therapies are introduced [1,2]. These therapies, including hemodialysis and kidney transplant, are not only costly but will also lead to major health and social complications, led by dependencies on the procedure (hemodialysis) and immunosuppressive medications (kidney transplant). This will subsequently reduce the quality of life and create a burden in the healthcare system [3,4,5].

Over the past couple of decades, various treatments have been developed to delay the progression of CKD. The most effective method is by controlling blood pressure, namely using the angiotensin-converting enzyme inhibitors [6,7]. Other therapeutic approaches, such as the normalization of blood glucose and cholesterol and several lifestyle and diet changes, have also been applied [8]. However, none of these approaches has resulted in the reversal of the disease and there is no drug available that could improve kidney function in CKD patients [9]. Therefore, there is an urgent need to search for a new drug and develop a new therapy that can slow down or reverse the progression of CKD before it advances to potentially fatal ESRF.

Adenine-induced rodent models have been extensively used in previous studies to simulate CKD in humans [10,11]. Previous studies have shown that mice treated with 0.2% adenine for four weeks displayed morphological, biochemical and histopathological changes, as well as elevated levels of markers of inflammation, kidney injury, and oxidative and nitrosative stress, which correspond to what is observed in CKD cases in humans [12,13,14].

Catalpol (CAT) is an iridoid glucoside isolated from the root of *Rehmannia glutinosa*, a widely used herbs in traditional Chinese medicine and it has been used to manage diabetes for over a millennium [15]. Previous studies have shown that CAT possesses myriad therapeutic properties, including anti-inflammatory [16,17], antioxidant [18,19], cardioprotective [20], antiapoptotic [21], antihyperglycemic [22] and antifibrosis [23], in rodent models and cell cultures.

Since the pathogenesis of experimental and clinical CKD involves inflammatory and oxidative actions, and CAT manifests various therapeutic actions, including anti-inflammation and antioxidants [24,25], we thought that it would be compelling to evaluate the effectiveness of CAT in mitigating or preventing CKD in mice, which, to our knowledge, has not yet been reported. Therefore, the aim of our study is to evaluate the potential ameliorative effects of CAT in adenine-induced mice with CKD by evaluating several markers of inflammation, oxidative stress, deoxyribonucleic acid (DNA) damage and apoptosis. In addition to that, we wanted to explore the possible mechanisms involved by evaluating the expression of the proteins that regulate inflammation, phosphorylated nuclear factor-κB (phospho-NF-κB) as well as its antagonist sirtuin-1 [26].

## 2. Materials and Methods

### 2.1. Animals

Equal numbers of six- to eight-week-old male and female BALB/c mice (College of Medicine and Health Sciences animal house, UAEU, Al Ain, United Arab Emirates) weighing 25–30 g were housed in temperature-controlled (22 ± 1 °C) rooms and maintained on a 12-h light-dark cycle with a humidity of 50–60%. They had unrestricted access to drinking water and commercially available laboratory chow, which consisted of 24% protein, 2% fat, and 8% fiber (National Feed and Flour and Marketing Co., Abu Dhabi, United Arab Emirates).

### 2.2. Experimental Design

The mice were indiscriminately segregated into four groups (n = 8), with two groups receiving 0.2% adenine from Sigma Aldrich Co. (St. Louis, MO, USA) mixed with powdered feed for four weeks. Two groups of mice were given an oral gavage treatment of CAT, purchased from Sigma Aldrich Co. (St. Louis, MO, USA), daily for the duration of four weeks, except on weekends. We referred to previous publications to determine the dose of adenine for this study [27], and the dose selection of CAT was done based on previous studies where it has demonstrated anti-inflammatory and antioxidant activities [20,25].

The treatment of the mice was done as follows:

- The control group was given standard feed along with saline (10 mL/kg) via oral gavage.
- The adenine group was given the same standard diet in powdered form containing 0.2% adenine (0.2 g of adenine in 100 g of powdered feed) with saline (10 mL/kg) via oral gavage.
- The CAT group was given standard feed with CAT (5 mg/kg, dissolved in saline) via oral gavage.
- The CAT + adenine group was given 0.2% adenine prepared similar to the adenine group with CAT (5 mg/kg, dissolved in saline) via oral gavage.

The mice were sacrificed after four weeks, and one day prior to that, they were placed in metabolic cages for 24 h to measure urine output and water intake. The collected urine was stored at −80 °C pending further analysis.

The body weight of the animals was documented at the beginning of the study and immediately before sacrifice. Instantly after urine collection, mice were given sodium pentobarbital (60 mg/kg) intraperitoneally, and blood was collected via the inferior vena cava into a tube containing sodium citrate (4%). The blood was then centrifuged for 15 min at 900× *g* and 4 °C, and the plasma obtained was kept at −80 °C for future analysis. Both kidneys were extracted and weighed. A small piece of the upper part of the right kidney was cut and fixed in 10% formalin for histopathological analysis (n = 6), whereas the remaining right and left kidneys were wrapped and dipped in liquid nitrogen and kept frozen at −80 °C for further analysis.

### 2.3. Homogenization of the Kidney

The kidney tissues were homogenized in 2 mL homogenization vials along with potassium chloride buffer added with protease and phosphatase inhibitor cocktails and 2.0 mm zirconia beads from BioSpec (Bartlesville, OK, USA). The homogenization was done using the Precellys homogenizer from Bertin Instruments (Bretonneux, France) for two times of three cycles of 10 s at 6500× *g*, and the homogenates were centrifuged at 14,000× *g* for 20 min. The supernatants were divided into four aliquots and frozen at −80 °C until further analysis. The protein estimation was done using the Pierce™ BCA Protein Assay Kit from Thermo Scientific (Rockford, IL, USA), as per the protocol provided by the manufacturer.

### 2.4. Biochemical Parameters

The concentrations of creatinine, urea and albumin were quantified using commercially available kits from Roche Diagnostics (Indianapolis, IN, USA).

### 2.5. Measurement of Markers of Kidney Injury in Plasma

The levels of kidney injury molecule-1 (KIM-1), neutrophil gelatinase-associated lipocalin (NGAL), and adiponectin and cystatin C in plasma were measured using ELISA kits from R&D Systems (Minneapolis, MN, USA).

### 2.6. Assessment of Thiobarbituric Acid Reactive Substances (TBARS) and Catalase Activity in Kidney Homogenates

The level of lipid peroxidation was evaluated by measuring its byproduct, TBARS, using malondialdehyde (MDA) from Sigma Aldrich Co. (St. Louis, MO, USA) to generate a standard curve [28]. The activity of the catalase was determined according to the protocol provided by Cayman Chemicals (Ann Arbor, MI, USA).

### 2.7. Measurement of Markers of Inflammation in Kidney Homogenates

The concentrations of proinflammatory cytokines, including tumor necrosis factor α (TNFα), and interleukin (IL)-6 in kidney homogenates were determined using ELISA kits from R&D Systems (Minneapolis, MN, USA).

### 2.8. DNA Damage Analysis by Comet Assay

In a separate set of mice (n = 5), the kidneys of the mice were extracted following sacrifice and immediately processed to evaluate the DNA damage as per the standard Comet assay protocol [29,30]. The DNA damage was assessed by measuring the length of DNA migration, which included the diameter of the nucleus and the migrated DNA, using image analysis Axiovision 3.1 software by Carl Zeiss (White Plains, NY, USA).

### 2.9. Assessment of the Levels of Procaspase-3 and Cleaved Caspase-3 in Kidney Homogenates

The level of procaspase-3 was quantified using a colorimetric protease assay kit from Invitrogen (Waltham, MA, USA). The level of cleaved caspase-3, a key mediator of apoptosis, was assessed using an ELISA kit from R&D Systems (Minneapolis, MN, USA).

### 2.10. Western Blot Analysis of NF-κB and Phospho-NF-κB in Kidney Homogenates

The protein expressions of the NF-κB and phospho-NF-κB in kidney homogenate were obtained using the Western blot method. Thirty micrograms of kidney homogenate was separated electrophoretically in 10% sodium dodecyl sulfate polyacrylamide gel and subsequently transferred onto polyvinylidene difluoride membranes. The blots were then blocked with 5% bovine serum albumin for an hour at room temperature (RT) and probed overnight at 4 °C with 1:1000 dilution of mouse monoclonal NF-κB and phospho-NF-κB antibodies from Santa Cruz Biotechnology (Dallas, TX, USA). Next, a 1:10,000 dilution of rabbit anti-mouse IgG from Abcam (Boston, MA, USA) was used to incubate the blots at RT for 2 h. The development of the blots was done using the SuperSignal™ West Pico PLUS chemiluminescent substrate from Thermo Scientific (Rockford, IL, USA) and protein band density was measured using the image processing program ImageJ (Bethesda, MD, USA). The blots were then incubated with a 1:10,000 dilution of mouse monoclonal GAPDH or β-actin antibodies from Abcam (Boston, MA, USA), and the band density of these proteins was used as the endogenous controls.

### 2.11. Measurement of the Concentration of Sirtuin-1 in Kidney Homogenates

The concentration of sirtuin-1 in kidney homogenates was determined using a commercially available ELISA kit from MyBiosource (San Diego, CA, USA).

### 2.12. Histopathological Analysis

As per the standard histological protocol, the samples (n = 6 in each group) were fixed, grossed, processed and embedded in paraffin [31]. Then, they were sectioned into 4 µm pieces using a microtome from Leica Biosystems (Nussloch, Germany) and mounted onto slides. The slides were stained with hematoxylin and eosin (H&E) to assess acute tubular necrosis, Sirius red stain to evaluate interstitial fibrosis, and periodic acid Schiff (PAS) stain to assess the glomerular integrity [32,33]. The stained sections were blindly evaluated under a light microscope by a pathologist. The scoring was performed semi-quantitatively based on the percentage of renal tubular necrosis. A score of 0 was given to a slide that exhibited normal kidney architecture with no tubular injury, a score of 1 for tissues that had less than 25% tubular injury, 2 for tissues with tubular injury of 26–50%, 3 for 51–75%, and a score of 4 was given to tissues with more than 75% of necrotic tubular injury. The proportion of red-stained fibrotic area in the cortex of each section, stained with Sirius red stain, was graded semi-quantitatively and the fibrosis index was calculated and expressed in percentage according to the protocol previously reported [32,34].

### 2.13. Statistics

The statistical analysis for this study was done using GraphPad Prism Software 7 (San Diego, CA, USA). Comparison between studied groups (Control vs. Adenine, Control vs. CAT, Adenine vs. CAT + Adenine, and CAT vs. CAT + Adenine) was done using the one-way analysis of variance and Holm-Sidak’s multiple comparison test. Data in the figures were expressed as mean ± SEM and *p* values < 0.05 were considered to be statistically significant.

## 3. Results

### 3.1. The Effects of CAT on Physiological and Biochemical Parameters

Table 1 shows that mice treated with 0.2% adenine for the duration of four weeks had a significant reduction in body weight (*p* < 0.0001). Conversely, the kidney weight, water intake and urine volume were significantly increased in the adenine group compared with the control (*p* < 0.0001, *p* < 0.0001, and *p* < 0.0001, respectively). A significant ameliorative effect was observed when CAT treatment was given along with adenine for body weight change (*p* < 0.01), kidney weight change (*p* < 0.0001), water intake (*p* < 0.0001) and urine volume (*p* < 0.0001), when compared with the adenine-treated group.

Our biochemical tests showed significant elevation in the levels of urea (*p* < 0.0001) and creatinine (*p* < 0.0001) in plasma among adenine-treated animals compared with the control. However, when CAT was given in tandem with adenine, the levels of these biomarkers were significantly reduced compared to the adenine group (*p* < 0.0001 and *p* < 0.0001; Table 2). Adenine treatment caused a significant reduction in creatinine clearance (*p* < 0.001), and a significant increase was observed in the CAT + adenine group when compared with the adenine-treated animals (*p* < 0.05, Table 2). Table 2 also shows that adenine caused a significant surge in the albumin and creatinine ratio (*p* < 0.0001), and an opposing effect was observed when adenine-treated mice were given CAT (*p* < 0.0001).

### 3.2. The Effects of CAT on the Concentrations of Markers of Kidney Injury in Plasma

**Figure 1:**
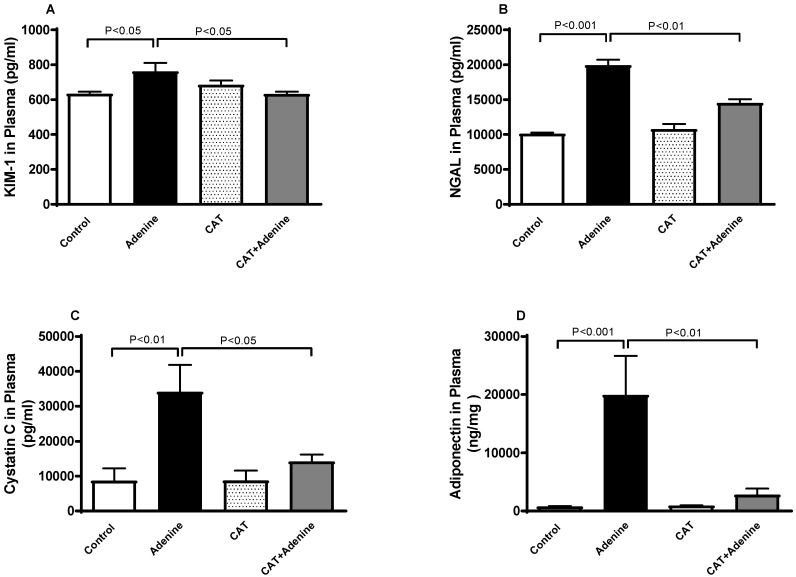
Plasma concentrations of kidney injury molecule-1 (KIM-1, (**A**)), neutrophil gelatinase-associated lipocalin (NGAL, (**B**)), cystatin C (**C**) and adiponectin (**D**) in mice with or without adenine (0.2% *w*/*w*, for 4 weeks) added to the feed and oral treatment of saline or catalpol (CAT, 5 mg/kg) for 4 weeks except during the weekends. Mean ± SEM (n = 8). Statistical analysis by one-way analysis of variance followed by Holm Sidak’s test.

Figure 1 depicts the effect of CAT on various markers of kidney impairment. Adenine caused an elevation at a significant proportion in the plasma levels of KIM-1, NGAL, cystatin C and adiponectin (*p* < 0.05, *p* < 0.001, *p* < 0.01 and *p* < 0.001, respectively). When CAT treatment was given concomitantly with adenine, the levels of KIM-1, NGAL, cystatin C and adiponectin showed significant reductions (*p* < 0.05, *p* < 0.01, *p* < 0.05, and *p* < 0.01, respectively) compared with the group treated with adenine alone.

### 3.3. The Effects of CAT on Lipid Peroxidation and Antioxidant Levels in Kidney Homogenates

**Figure 2:**
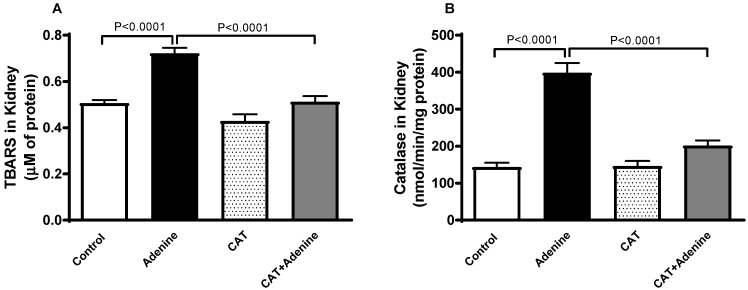
The concentration of thiobarbituric acid reactive substances (TBARS, (**A**)) and the activity of catalase (**B**) in the kidney homogenate of mice with or without adenine (0.2% *w*/*w*, for 4 weeks) added to the feed and oral treatment of saline or catalpol (CAT, 5 mg/kg) for 4 weeks, except during the weekends. Mean ± SEM (n = 8). Statistical analysis by one-way analysis of variance followed by Holm Sidak’s test.

The concentration of TBARS, a marker of lipid peroxidation, was significantly elevated in the adenine group (*p* < 0.0001) when compared with the control group. This elevation was significantly reduced when treatment with CAT was administered concurrently (*p* < 0.0001). Similarly, a significant surge was documented in catalase activity in adenine-treated mice compared to the control (*p* < 0.0001). This effect was markedly reduced (*p* < 0.0001) with CAT pretreatment (Figure 2).

### 3.4. The Effects of CAT on the Levels of Proinflammatory Cytokines in Kidney Homogenates

**Figure 3:**
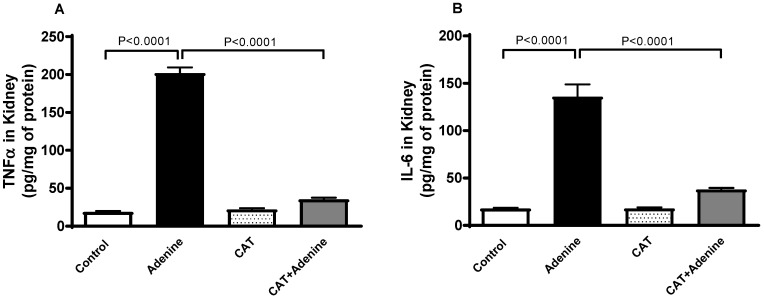
The concentrations of tumor necrosis factor alpha (TNFα, (**A**)) and interleukin 6 (IL-6, (**B**)) in kidney homogenate of mice with or without adenine (0.2% *w*/*w*, for 4 weeks) added to the feed and oral treatment of saline or catalpol (CAT, 5 mg/kg) for 4 weeks, except during the weekends. Mean ± SEM (n = 8). Statistical analysis by one-way analysis of variance followed by Holm Sidak’s test.

Adenine-treated mice demonstrated a significant elevation in the kidney concentrations of proinflammatory markers TNFα and IL-6 compared to the control mice (*p* < 0.0001 and *p* < 0.0001, respectively, Figure 3). However, these elevations were significantly mitigated when CAT was given alongside adenine (*p* < 0.0001 and *p* < 0.0001).

### 3.5. The Effects of CAT on Kidney DNA Damage

**Figure 4:**
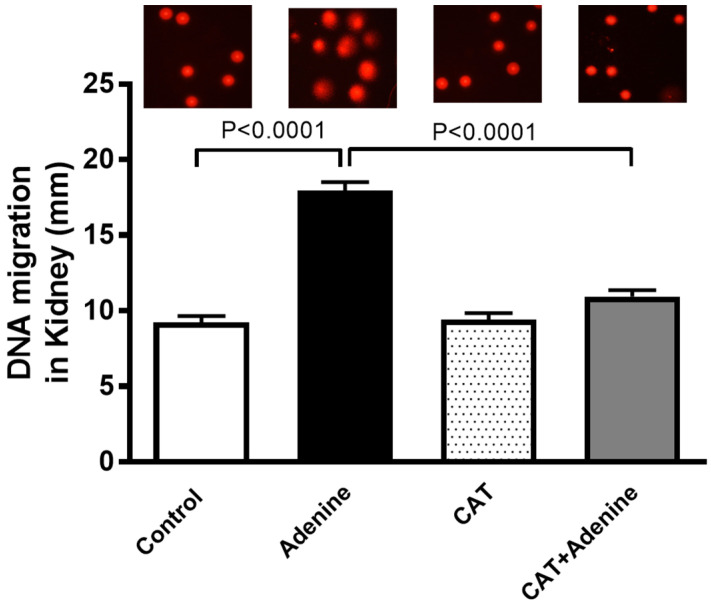
DNA migration (mm) in kidney tissue by Comet assay in mice with or without adenine (0.2% *w*/*w*, for 4 weeks) added to the feed and oral treatment of saline or catalpol (CAT, 5 mg/kg) for 4 weeks, except during the weekends. Mean ± SEM (n = 5). Statistical analysis by one-way analysis of variance followed by Holm Sidak’s test.

As depicted in Figure 4, the adenine-treated group exhibited a significant increase in DNA damage in the kidney tissue when compared with the control group (*p* < 0.0001). Nevertheless, a significant preventive effect was observed when CAT was given to the adenine-treated mice (*p* < 0.0001).

### 3.6. The Effects of CAT on the Levels of Procaspase-3 and Cleaved Caspase-3 in Kidney Homogenate

**Figure 5:**
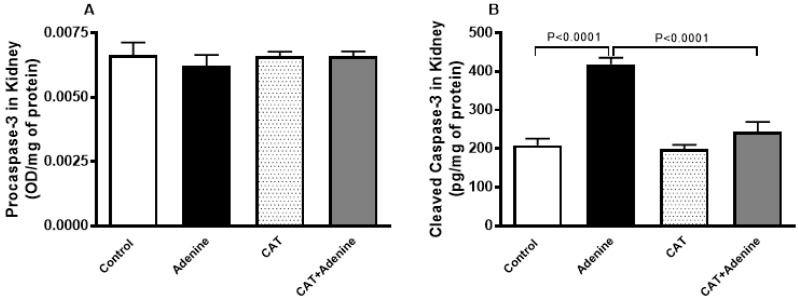
The levels of procaspase-3 (**A**) and cleaved caspase-3 (**B**) in the kidney homogenate of mice with or without adenine (0.2% *w*/*w*, for 4 weeks) added to the feed and oral treatment of saline or catalpol (CAT, 5 mg/kg) for 4 weeks, except during the weekends. Mean ± SEM (n = 8). Statistical analysis by one-way analysis of variance followed by Holm Sidak’s test.

Figure 5 shows that while the level of procaspase 3 was slightly but statistically insignificantly decreased, the level of cleaved caspase-3 in the kidney was significantly higher in the adenine group compared with the control group (*p* < 0.0001) and this increase was significantly diminished in mice treated with both adenine and CAT (*p* < 0.0001).

### 3.7. The Effects of CAT on the Expression of NF-κB and Phospho-NF-κB in Kidney Homogenate

**Figure 6:**
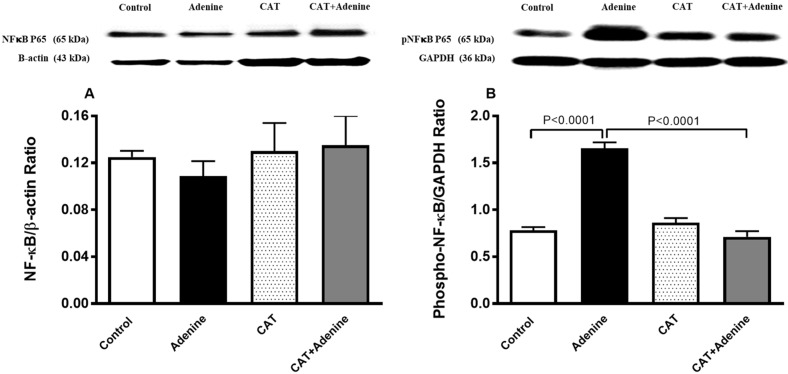
The nuclear factor-κB (NF-κB; (**A**)) and phosphorylated NF-κB (phospho-NF-κB; (**B**)) expressions in kidney homogenate of mice with or without adenine (0.2% *w*/*w*, for 4 weeks) added to the feed and oral treatment of saline or catalpol (CAT, 5 mg/kg) for 4 weeks, except during the weekends. Mean ± SEM (n = 8). Statistical analysis by one-way analysis of variance followed by Holm Sidak’s test.

Figure 6 shows that treatment with adenine induced an insignificant reduction in the expression of NF-κB. Nevertheless, a notable and statistically significant surge in the expression of phospho-NF-κB in the kidney homogenate was observed in the adenine group compared with the control group (*p* < 0.0001, Figure 6). This surge was significantly prevented by treatment with CAT (*p* < 0.0001).

### 3.8. The Effects of CAT on the Concentrations of Sirtuin-1 in Kidney Homogenate

**Figure 7:**
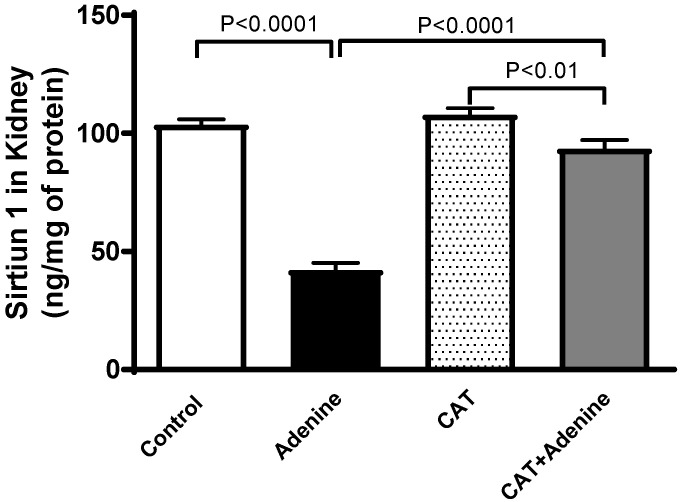
The concentration of sirtuin-1 in kidney homogenate of mice with or without adenine (0.2% *w*/*w*, for 4 weeks) added to the feed and oral treatment of saline or catalpol (CAT, 5 mg/kg) for 4 weeks, except during the weekends. Mean ± SEM (n = 8). Statistical analysis by one-way analysis of variance followed by Holm Sidak’s test.

Figure 7 depicts that the level of sirtuin-1 was significantly reduced in the adenine group compared to the control (*p* < 0.0001). This reduction, however, was significantly prevented in mice treated with both CAT and adenine when compared with the adenine-only group (*p* < 0.0001).

### 3.9. Histopathological Findings

**Figure 8:**
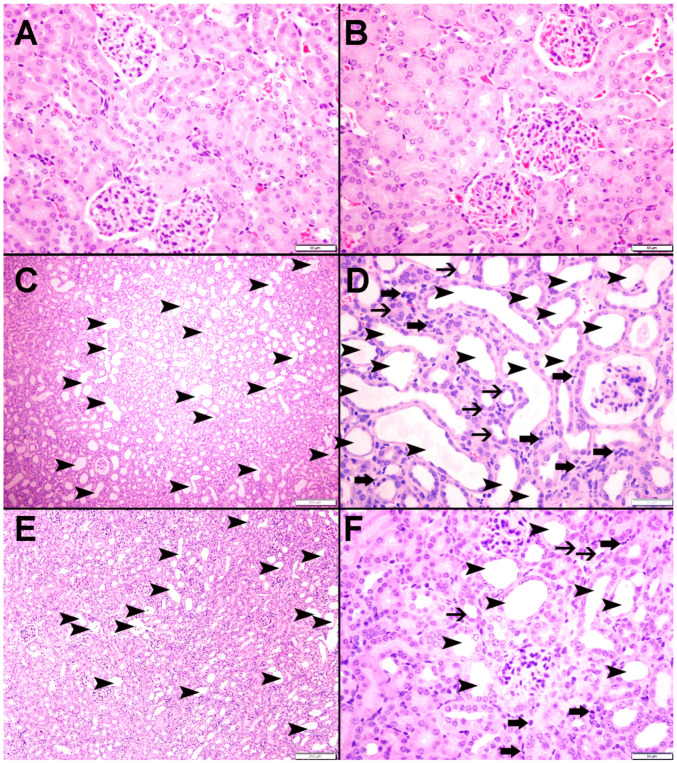
The representative light microscopy sections of kidney tissue of mice with or without adenine (0.2% *w*/*w*, for 4 weeks) added to the feed and oral treatment of saline or catalpol (CAT, 5 mg/kg) for 4 weeks except during the weekends. The Control (**A**) and CAT-treated (**B**) groups showed normal kidney architecture and histology. The adenine-treated group (**C**,**D**) showed large areas of dilated tubules (arrowheads), foci of tubular atrophy (thin arrows) and interstitial lymphocyte infiltration (thick arrows). The CAT + adenine group (**E**,**F**) showed a significant ameliorative effect with a reduction in the foci of dilated tubules (arrowheads), foci of tubular atrophy (thin arrows) and interstitial lymphocyte infiltration (thick arrows).

Figure 8 illustrates representative images of the H&E staining of the kidney tissues. The control (Figure 8A) and CAT (Figure 8B) groups displayed normal kidney architecture with intact glomeruli and renal tubules; therefore, they were scored 0. The adenine-treated group (Figure 8C,D) was scored 2, as they exhibited foci of tubular injury with tubular injury in 48.2 ± 3.96% of the examined tissue areas, loss of brush border of proximal tubules, and mixed inflammatory cell infiltration of the interstitium consisting of lymphocytes. However, mice treated with both CAT and adenine (Figure 8E,F) showed a significant alleviation in the damaging effect of adenine. The focal tubular injury with tubular dilatation was seen in 23.2 ± 2.68% (*p* < 0.0001 vs. adenine group) of the cortical tissue, and was scored 1.

**Figure 9:**
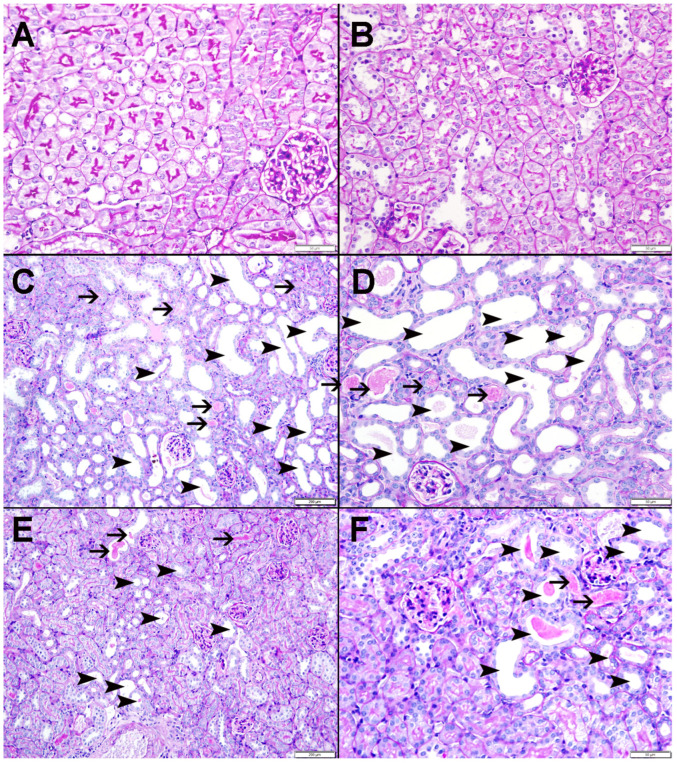
Representative images of periodic acid-Schiff (PAS) staining of kidney tissue of mice with or without adenine (0.2% *w*/*w*, for 4 weeks) added to the feed and oral treatment of saline or catalpol (CAT, 5 mg/kg) for 4 weeks, except during the weekends. The Control (**A**) and CAT-treated (**B**) groups showed normal kidney architecture and histology. The adenine-treated group (**C**,**D**) shows large areas of dilated tubules (arrowheads) and foci of acute tubular necrosis (thin arrows). The CAT + adenine group (**E**,**F**) showed a significant ameliorative effect with a reduction of foci of both dilated tubules (arrowheads) and acute tubular necrosis (thin arrows).

**Figure 10:**
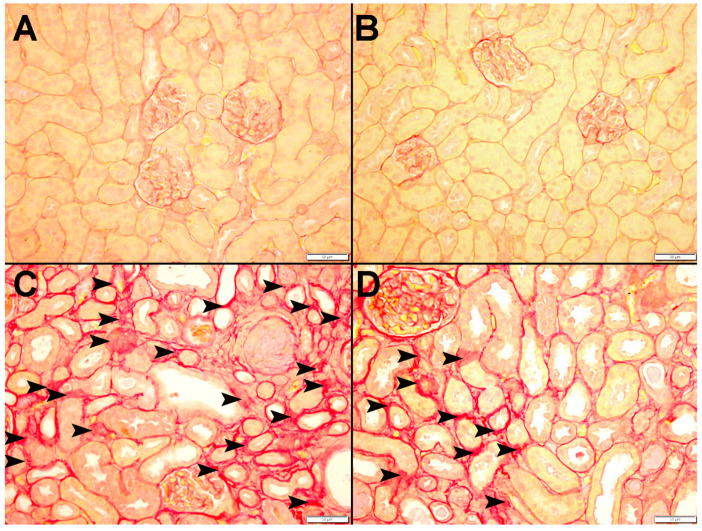
Representative images of Sirius red staining of kidney tissue of mice with or without adenine (0.2% *w*/*w*, for 4 weeks) added to the feed and oral treatment of saline or catalpol (CAT, 5 mg/kg) for 4 weeks, except during the weekends. The Control (**A**) and CAT-treated (**B**) groups showed normal kidneys. The adenine-treated group (**C**) showed a large area of interstitial fibrosis (arrowhead). The CAT + adenine group (**D**) showed a significant ameliorative effect with focal interstitial fibrosis (arrowhead) interstitial fibrosis.

Figure 9 and Figure 10 display representative images of the PAS and Sirius red staining of the kidney tissues. The control (Figure 9A and Figure 10A) and CAT (Figure 9B and Figure 10B) exhibited normal kidney architecture and histology, with no increase in interstitial fibrosis and were given a score of 0. The adenine group (Figure 9C,D and Figure 10C) exhibited focal tubular atrophy with foci of acute tubular necrosis with interstitial fibrosis of 32.8 ± 2.99% of the cortical tissue and was scored 2. Meanwhile, the CAT + adenine group (Figure 9E,F and Figure 10D) displayed foci of dilated tubules with foci of acute tubular necrosis and a significant ameliorative effect, with interstitial fibrosis in only 22.6 ± 2.54% (*p* < 0.01 vs. adenine group) of the cortical tissue and was given a score of 1.

## 4. Discussion

In the present study, we showed that daily oral treatment with 5 mg/kg CAT has significantly ameliorated CKD-associated biochemical, physiological and histopathological alterations without causing any detrimental side effects.

CKD is an insidious and multicausal disease that, regardless of the cause, has common characteristics of repeated glomerular and tubular injury associated with the elevation of the endogenous reactive oxygen species (ROS). The upregulation of ROS in turn leads to inflammation, oxidative stress, DNA damage, changes in cell proliferation and repair, and cell death, thereby resulting in fibrosis, a major indicator of CKD [14,35,36,37]. Subsequently, the presence and accumulation of fibrosis in the kidney causes irreversible nephrotic loss and leads to the progression of CKD to ESRD [7,36]. Most conventional drugs and approaches to CKD today, however, are effective at delaying but not at preventing its deterioration [8,38].

In the present work, we applied a method that has been extensively utilized in the past to induce CKD in rodent models [13]. Treatment of mice with 0.2% adenine over a period of four weeks has been shown to mimic the biochemical, physiological and histological changes observed in CKD in humans [5,12,39]. The orally administered adenine is metabolized to a low-soluble 2,8-dihydroxyadenine, which deposits and forms tubular crystals and thus causes kidney damage. The occlusion of renal tubules induced by the crystals inhibits the excretion of nitrogenous substances, leading to biochemical and physiological changes that resemble CKD in humans [5,12,39]. The latter makes it a biologically plausible model for studying the nephroprotective effects of CAT [5,12,39]. We have treated our adenine-induced CKD models with 5 mg/kg CAT, and observed that animals treated with CAT on their own did not show any adverse effect or have altered parameters that were assessed in this study. The compound and dose were selected based on their efficacy in ameliorating inflammatory, oxidative and apoptosis events in various animals and cell models of diabetic nephropathy, endometritis, neurogenerative disorder and cardiomyopathy [16,17,18,40].

Treatment with adenine has caused physiological changes, including a significant reduction in body weight, as well as a significant increase in kidney weight, water intake, and urine volume, comparable to previously reported studies [41,42,43]. The decrease in body weight is in line with cachexia observed in CKD patients [44], whereas increased urine output might have to do with the reduced efficiency of the kidney to filter blood and to reabsorb water [45], which subsequently leads to an increase in water consumption. The increase in kidney weight is possibly due to the buildup of fibrotic tissue in the kidney. The latter is driven by a repair response, characterized by the accumulation of extracellular matrix in the glomeruli, and is a pathognomonic symptom of CKD [46]. Moreover, we have also confirmed that, as per previous reports [5,8,13], the reduction of kidney function in the adenine-treated mice is characterized by a significant reduction in creatinine clearance, elevation of plasma concentration of urea, and urinary concentrations of creatinine and albumin, which are in line with CKD cases in humans [47,48]. These physiological and biochemical parameters were significantly normalized with the pretreatment of CAT.

Markers customarily used to detect kidney injuries associated with CKD, such as KIM-1, NGAL, cystatin C and adiponectin were also assessed in our study [8,49,50]. These biomarkers of reduced renal function were found to be significantly elevated in adenine-treated mice, which is in line with previous reports [51,52,53] and were reduced markedly when treatment with CAT was given concomitantly with adenine.

The overproduction of ROS and the inadequate response of antioxidants play an important role in the pathogenesis of CKD, both clinically and experimentally [14,32,36,54]. Due to its reactivity to lipids, ROS oxidize the lipids from cell membranes, causing lipid peroxidation, which eventually leads to the formation of reactive aldehydes, namely MDA, a bioactive marker that was measured in our study [36,54]. Treatment with adenine significantly increased the concentration of MDA, which corroborates previous experimental and clinical studies [39,55], and this action was significantly reversed by CAT. Furthermore, we have observed a significant elevation in catalase activity, an antioxidant enzyme responsible for eliminating excess ROS, in adenine-treated mice, signifying that oxidative stress events are happening and that the compensatory process is occurring in the kidney to alleviate the detrimental effects of ROS [12,56]. Our findings indicated that CAT pretreatment significantly reduced the activity of this antioxidant. In addition to causing oxidative stress, the overproduction of ROS also plays a pivotal role in inflammatory responses [52]. It has been previously reported that adenine-treated mice exhibit elevated concentrations of proinflammatory cytokines in the kidney [8,11,43]. A similar pattern was observed in our study and was abrogated when CAT was given in parallel with adenine, hence supporting the reports that in addition to being an antioxidant, CAT is also a potent anti-inflammatory agent [20,40]. In fact, it has been previously reported that treatment with CAT ameliorated inflammation and oxidative stress in vivo in a myocardial reperfusion injury model and in doxorubicin-induced injury of H9C2 cardiomyocytes in vitro [20,40].

The loss of equilibrium in the redox balance is fundamental to CKD progression. In addition to lipids and proteins, excess ROS can also oxidize DNA, causing DNA damage [57]. Many of the oxidative DNA damage might not be repaired due to the inability of the compensatory mechanism to cope with the severity of the damage [58,59]. The occurrence of DNA damage in the peripheral blood lymphocytes of CKD patients has been reported in several studies [60,61]. In the present study, we found a significant increase in DNA damage in the kidneys of the adenine-treated group. This effect was abrogated in mice treated with CAT and adenine. Our data corroborate a recent study that showed that CAT prevented DNA damage in the hearts of mice exposed to diesel exhaust particles [25].

It is well known that unrepaired DNA damage can induce various cascades of responses, including cell death, mediated by different mechanisms, including apoptosis. The latter is a programmed cell death modulated by the activation of caspases, including the executioner caspase 3 [62]. Here, we found that adenine induced a significant increase in the concentration of cleaved caspase-3, the active form of caspase-3. Compellingly, our data elucidated that pretreatment with CAT prevented the event of apoptosis, marked by a significant reduction in cleaved caspase-3 in the kidney. Our results are in line with previous reports, which showed that CAT significantly reduced the expression of cleaved caspase-3 in a mouse model of myocardial reperfusion injury [20], as well as other apoptotic markers, including Bax and BCL-2, in murine models of diabetic nephropathy and diabetes mellitus-induced male reproductive damage [21,24].

NF-κB is involved in myriads of pathogenesis of inflammatory diseases, including kidney disease [63]. In the absence of a cellular stimulant, NF-κB resides in the cytoplasm and is inhibited by the IκB proteins. The increase of ROS or in the presence of other stimulants, IκB will be phosphorylated and degraded, which liberates the NF-κB. The NF-κB then migrates to the nucleus, where it is phosphorylated at the p65 subunit [26,64,65]. The latter event subsequently activates the transcription of inflammatory cytokines [65,66]. Therefore, we evaluated the expression of NF-κB in the kidney to elucidate its role in the anti-inflammatory mechanism of CAT. Our data showed that the administration of adenine significantly elevated the kidney concentration of phospho-NF-κB, and CAT treatment significantly reversed this effect. These findings corroborate recent studies that showed that pretreatment with CAT mitigated inflammatory actions in mice with chronic inflammatory skin disease, endometritis and cardiac inflammation via the inhibition of the NF-κB pathway [16,25].

To gain more insight into the mechanisms involved in the nephroprotective effects of CAT, we evaluated the concentration of sirtuin-1 in the kidneys. Sirtuin-1 regulates mammalian oxidative respiration, anti-inflammatory responses, and redox signaling pathways; the latter is critical in regulating the pathogenesis of various diseases, including kidney diseases [26,37,67]. Studies have suggested that ROS control and cause an inhibitory effect on sirtuin-1 by oxidizing its cysteine residue. The inhibition of sirtuin-1 enhances the NF-κB signaling and subsequently activates inflammatory mechanisms [68]. As demonstrated in previous reports, the relationship between sirtuin-1 and NF-κB is antagonistic [26]. Sirtuin-1 obstructs the NF-κB activity by deacetylating its p65 subunit, leading to the inhibition of the inflammation signaling cascade [26]. The concentration of sirtuin-1 has been reported to be downregulated in chronic inflammation and oxidative stress [37,69], an observation that is concurred with our study, where mice that exhibited elevated inflammatory and oxidative stress parameters related to adenine-induced CKD showed a significant reduction in the level of sirtuin-1. This effect was significantly reversed with the treatment of CAT, which corroborates recent studies, where the ameliorative effect of CAT is linked to its ability to activate sirtuin-1 in mice with chronic skin disease [70], as well as rats with polycystic ovarian syndrome and colitis [71,72].

The downregulation of sirtuin-1 and the increase in inflammation have been associated with the increased formation of tubular fibrosis in CKD [37,73]. This is in line with our histological observations. Adenine-treated mice showed significant elevation in necrotic and fibrotic incidences, as well as tubular dilation in the kidney, which was significantly ameliorated with the pretreatment of CAT. This mitigating effect may be in part due to the activation of sirtuin-1, which inhibits not only the inflammatory signals but also TGF- β and other fibrotic mediators [73]. This inhibition subsequently attenuates the formation of fibrotic tissues and corroborates a previous study on the effectiveness of CAT in relieving liver fibrosis in rodents [23].

## 5. Conclusions

In conclusion, our data demonstrated that CAT exhibited ameliorative effects against adenine-induced CKD in mice by mitigating inflammation and oxidative stress, which subsequently suppressed the occurrences of DNA damage and apoptosis and consequently led to the reduction of kidney injury markers and fibrosis through mechanisms involving sirtuin-1 activation and NF-κB inhibition. There was no identifiably overt side effect with CAT treatment. Further investigations should be done in order to consider CAT as an efficacious agent for the clinical treatment of CKD.

## Figures and Tables

**Table 1:** Some physiological parameters of control mice and those with adenine-induced chronic kidney disease with or without catalpol (CAT) treatment.

Parameters/Treatment	Control	Adenine	CAT	CAT + Adenine
Body weight change (%)	5.56 ± 1.23	−28.35 ± 0.98 ***	2.68 ± 0.69	−15.84 ± 2.93 ^Δ ᴏᴏᴏ^
Kidney weight change (%)	1.07 ± 0.01	2.88 ± 0.13 ***	1.06 ± 0.02	1.57 ± 0.06 ^ΔΔΔ ᴏᴏ^
Water intake	8 ± 1.06	28.67 ± 1.35 ***	11.67 ± 0.61	23.17 ± 1.55 ^ΔΔΔ ᴏᴏᴏ^
Urine volume	3.61 ± 0.28	17.72 ± 1.56 ***	4.08 ± 0.21	9.83 ± 0.47 ^ΔΔΔ ᴏᴏ^

Values in the table are mean ± SEM (n = 6). *** *p* < 0.0001 in control vs. adenine group. ^Δ^ *p* < 0.01 and ^ΔΔΔ^ *p* < 0.0001 in adenine vs. CAT + adenine group. ^ᴏᴏ^ *p* < 0.001 and ^ᴏᴏᴏ^ *p* < 0.0001 in CAT vs. CAT + adenine group. Statistical analysis by one-way analysis of variance followed by Holm Sidak’s test. Adenine was added to the feed (0.2% *w*/*w*, for 4 weeks) and catalpol (CAT) treatment was given by oral gavage (5 mg/kg) on a daily basis for 4 weeks except during the weekends.

**Table 2:** The concentrations of urea and creatinine in plasma and creatinine clearance and albumin and creatinine ratio in control mice and those with adenine-induced chronic kidney.

Parameters/Treatment	Control	Adenine	CAT	CAT + Adenine
Urea (mmol/L)	4.12 ± 0.09	16.67 ± 0.52 ***	4.70 ± 0.23	7.28 ± 0.33 ^ΔΔΔ ᴏᴏᴏ^
Creatinine (µmol/L)	7.60 ± 0.48	19.42 ± 1.06 ***	7.83 ± 0.18	7.45 ± 0.63 ^ΔΔΔ^
Creatinine clearance (ml/min)	0.92 ± 0.11	0.20 ± 0.03 **	1.03 ± 0.13	0.62 ± 0.05 ^Δ ᴏ^
Albumin/creatinine (mg/mmol)	0.87 ± 0.12	20.87 ± 1.86 ***	1.03 ± 0.23	1.09 ± 0.06 ^ΔΔΔ^

Values in the table are mean ± SEM (n = 6). ** *p* < 0.001 and *** *p* < 0.0001 in control vs. adenine group. ^Δ^ *p* < 0.05 and ^ΔΔΔ^ *p* < 0.0001 in adenine vs. CAT + adenine group. ^ᴏ^ *p* < 0.05 and ^ᴏᴏᴏ^ *p* < 0.0001 in CAT vs. CAT + adenine group. Statistical analysis by one-way analysis of variance followed by Holm Sidak’s test. Adenine was added to the feed (0.2% *w*/*w*, for 4 weeks) and catalpol (CAT) treatment was given by oral gavage (5 mg/kg) on a daily basis for 4 weeks except during the weekends.

## Data Availability

The data presented in this study are available on request from the corresponding author.
